# A Rare Presentation of a Full-Thickness Rotator Cuff Tear With a Confluent Partial Articular-Sided Tear

**DOI:** 10.7759/cureus.8663

**Published:** 2020-06-17

**Authors:** Kee-Chi Daryl Oscar O Wong, Hamid Rahmatullah Bin Abd Razak, Tijauw-Tjoen Denny Lie

**Affiliations:** 1 Orthopaedics, Wollongong Hospital, Sydney, AUS; 2 Orthopaedic Surgery, Sengkang General Hospital, Singapore, SGP; 3 Musculoskeletal Sciences, Duke-Nus Medical School, Singapore, SGP; 4 Orthopaedic Surgery, Singapore General Hospital, Singapore, SGP

**Keywords:** rotator cuff tears, supraspinatus, clinician-measured outcomes, arthroscopic shoulder surgery

## Abstract

Rotator cuff tears (RCTs) are common injuries that often go undiagnosed. While there is strong literature addressing the management of both partial- and full-thickness tears of the supraspinatus, there is little to no literature describing both tears occurring concurrently. This is the first case reported in the context of a full-thickness RCT with a partial-thickness extension. It provides the clinical and radiographic context in which this patient was seen, as well as the operative guidelines used to achieve a successful outcome. We report a 39-year-old male reviewed for shoulder pain following mechanical injury. A tear involving the supraspinatus was clinically suspected and radiologically confirmed, but an unusual signal in select MRI images hinted towards further pathology. This was identified as a concurrent partial-thickness tear during arthroscopic evaluation was subsequently incorporated in the final repair configuration. The partial-thickness tear can easily be missed when superimposed on a full-thickness tear. A high degree of clinical suspicion is needed for diagnosis, corroborated with certain MRI features. Identifying these tears in a timely fashion will allow proper treatment to be instituted. Patients with these peculiar injury patterns can expect better long-term outcomes and functional recovery with proper diagnosis and treatment.

## Introduction

The rotator cuff is a group of four muscles originating from the scapula and inserting into the humerus. It primarily functions to provide dynamic stability to the glenohumeral joint [1]. The rotator cuff provides this stability by applying a centralizing force to allow the more superficial muscles to generate movement of the glenohumeral joint. Rotator cuff tears (RCTs) are a relatively common occurrence in the community, with more than half of documented cases persisting asymptomatically [2-6].

Supraspinatus tears are not uncommon in the community; a number of studies demonstrate incidence in more than 20% of an asymptomatic cohort [2-6]. Seitz describes that supraspinatus tears are a product of intrinsic (cellular ultrastructure, morphology, vascularity) and extrinsic factors (anatomical variation, scapular dyskinesia, postural abnormalities, muscle weakness) that result in tears in the distal muscle tendon, nearing the insertion on the superior aspect of the humeral head [7].

Supraspinatus tears have historically been described as full-thickness or partial-thickness tears, that is, a tear that extends through the full diameter of the tendon, or through a portion of its total cross-sectional area. These tears can be surgically repaired arthroscopically and have best results when achieved in a timely manner [8]. This case report describes the first documented sighting of an arthroscopic rotator cuff repair that revealed concurrent full-thickness and partial-thickness tears along the anterior and posterior bundles of the supraspinatus tendon. Given the paucity of published evidence in the identification and operative management of this presentation, we document journey of our patient from first consult, surgery, and finally follow-up and recovery.

## Case presentation

Preoperative consultation

**Figure 1:**
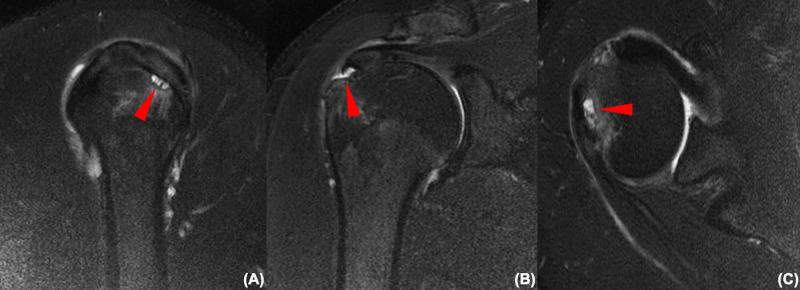
MRI images (A: sagittal view; B: coronal view; C: axial view) showing an obvious supraspinatus full-thickness tear as well as an increased signal inferi1or to the full-thickness tear (red arrows).

Our patient was a 39-year-old male of Asian ethnicity reporting a right shoulder injury in June 2017 after throwing a ball. This was initially managed by analgesia provided by his general practitioner, but the symptoms became recurrent and recalcitrant. During his first specialist outpatient appointment, he reported a visual analog scale (VAS) score of 6 out of 10. Functionally, the patient reported having difficulty in performing activities of daily living as well as moderate levels of pain aggravated by activity. Specific strength testing of the supraspinatus demonstrated weakness, and movements were globally limited. Special tests to elucidate rotator cuff pathology were positive, and the patient was referred for MRI of the right shoulder. MRI showed features of a full-thickness supraspinatus tear necessitating surgical repair. However, a number of slices also demonstrated an area of increased signal inferior to the described area of the original tear (Figure 1). This signal was not interpreted nor reported by the reading radiologist. Cuff muscles looked normal. The patient was prepped for surgery approximately one month following his injury.

Diagnostic arthroscopy

**Figure 2:**
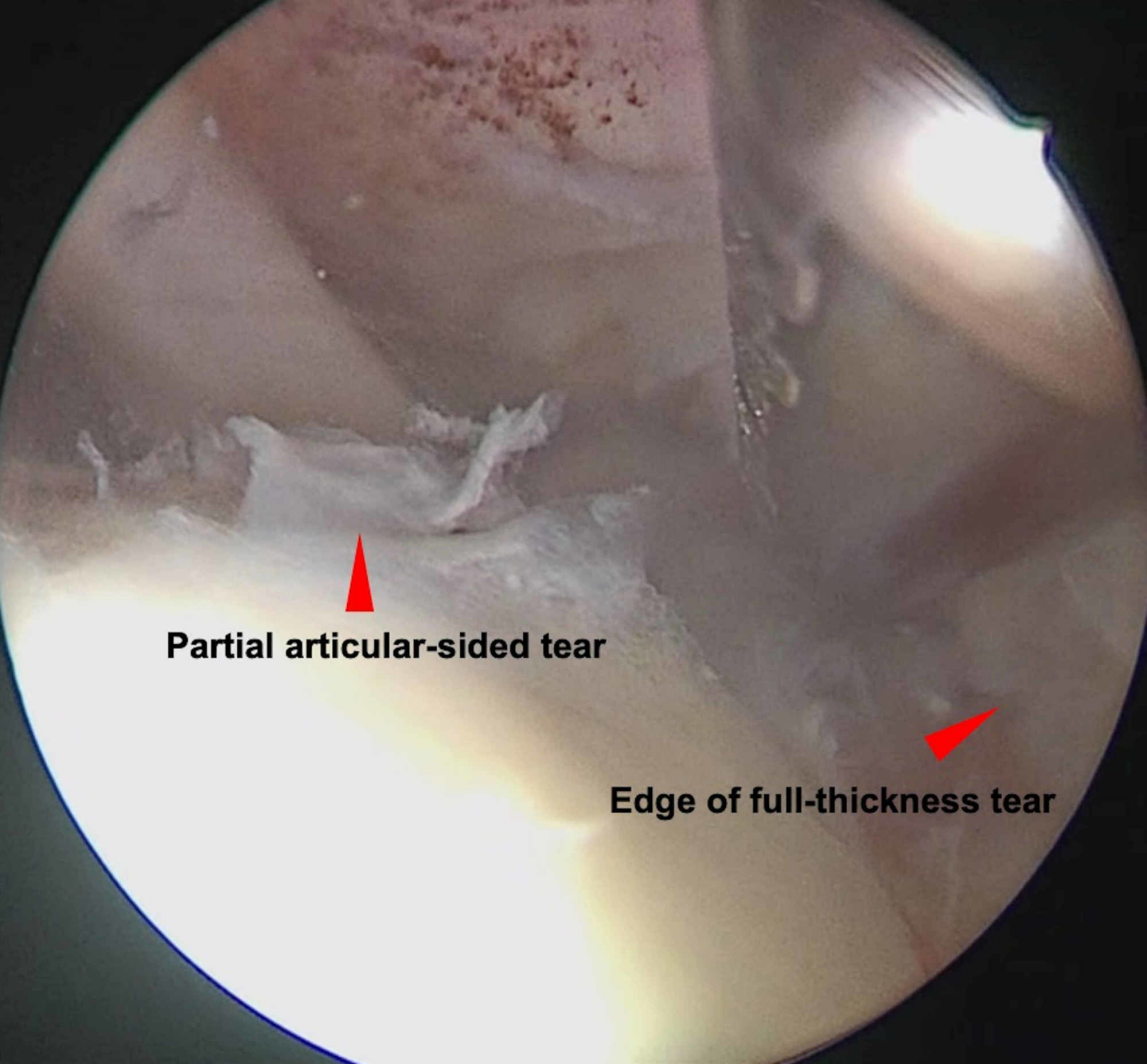
Glenohumeral arthroscopic view showing the full-thickness supraspinatus tear as well as the partial articular-sided tear in continuum (red arrows).

The patient was placed in a beach chair position under general anesthesia. Standard posterior and anterior arthroscopic portals were established. A mechanized fluid irrigation system was used. During diagnostic arthroscopy, a full-thickness tear was seen in the anterior bundle of the supraspinatus measuring 1.5 cm in the anteroposterior direction with minimal retraction. Additionally, a partial articular-sided tear was seen to extend posteriorly to the infraspinatus. The partial tear was continuous and connected to the full-thickness tear (Figure 2). The biceps tendon was found to be intact, as was the superior labrum. There was, however, extensive synovitis and bursitis in the subacromial space.

Surgical repair

Findings in the glenohumeral joint were noted and synovitis debrided. The tear in the anterior supraspinatus was seen and debrided via a direct lateral portal. The partial tear extending posterior was identified and debrided. A HEALIX TRANSTEND^TM ^(Depuy Mitek, Raynham, MA) implant system was used for the partial tear. A single-loaded anchor with two purple sutures was placed at the posterior end of the partial tear. This anchor was placed by piercing the intact portion of the partial tear. A triple-loaded 5.5-mm HEALIX ADVANCE anchor with six sutures was then placed at the footprint exposed by the full-thickness tear. The subacromial space was then entered and the bursitis debrided, carefully avoiding the sutures from the anchor.

One blue suture from the anterior triple-loaded anchor and one purple suture from the posterior single-loaded anchor were tied together to create a horizontal tie directly over and compressing the partial tear. The other blue suture from the anterior anchor was then passed antegrade to the midsubstance of the supraspinatus tendon. Similarly, the remaining purple suture from the posterior anchor was retrieved retrograde from the midsubstance of the tendon, just beside the blue suture. The paired tiger-striped sutures from the anterior anchor were then passed deep into the supraspinatus tendon medial to the blue and purple sutures placed earlier. Similarly, the remaining two purple sutures from the anterior anchor were passed into the supraspinatus tendon medial to the blue and purple sutures placed earlier.

**Figure 3:**
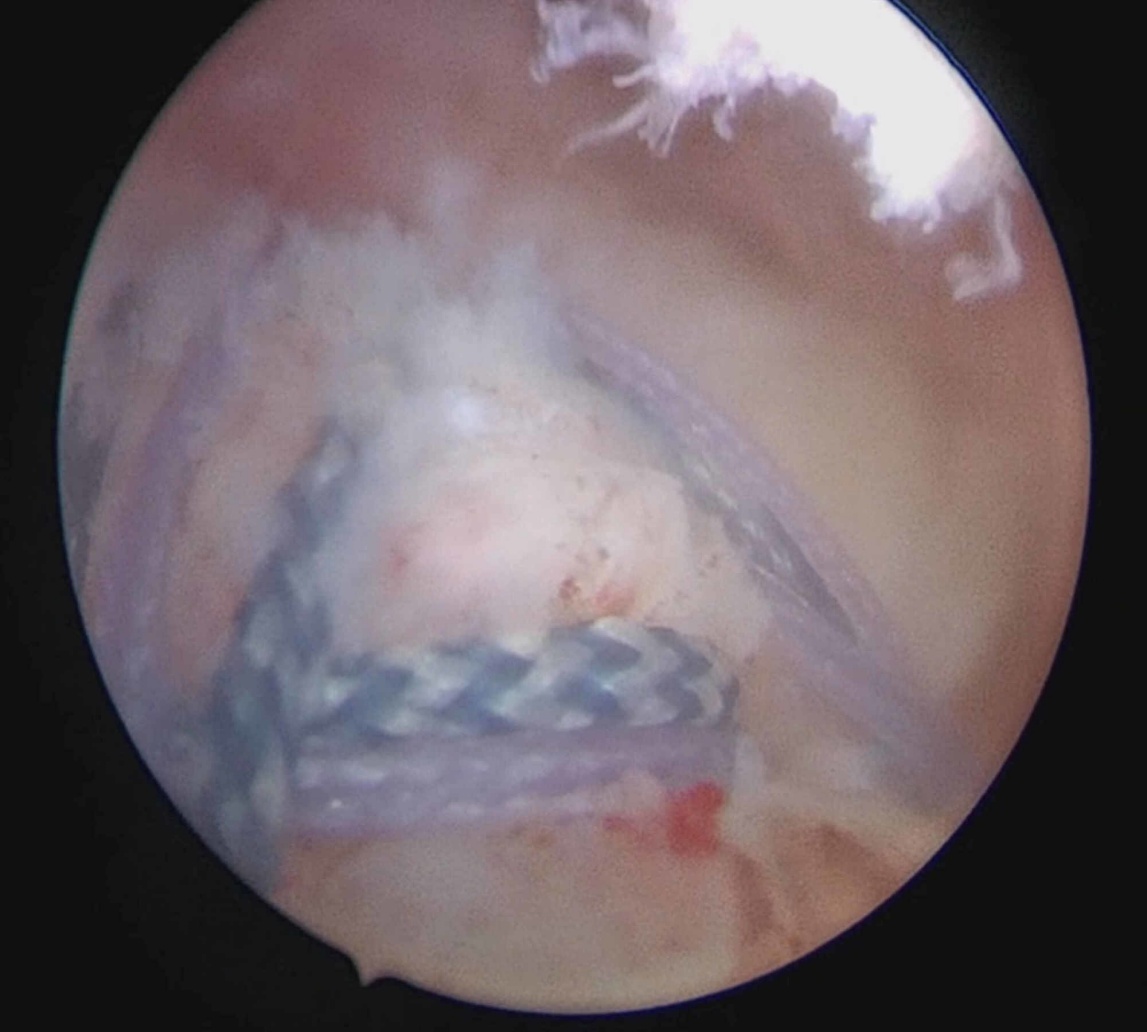
Subacromial view showing the final repair configuration incorporating both the full-thickness and partial-thickness tears via criss-crossing sutures from the suture anchor systems.

One tiger-striped suture and one purple suture from the anterior anchor, along with the purple suture from the posterior anchor, were then retrieved and fixed to a VERSALOK® (DePuy Mitek, Raynham, MA) suture anchor in the anterior greater tuberosity of the humoral head. Similarly, the remaining purple, tiger-striped, and blue sutures from the anterior Helix anchor were retrieved and secured with the VERSALOK suture anchor in the posterior greater tuberosity. This created a triangular dual row suture configuration, which was able to uniformly compress the tendon, addressing both the partial-thickness and full-thickness tears. As the horizontal tie was continuous to the purple and blue sutures secured to the lateral row, the tighter the sutures were pulled, the more compression was achieved onto the repair. This creates a stable, dynamic, and mutually supportive repair (Figure 3).

Postoperative care

The patient was satisfactorily discharged home on the first postoperative day. The patient was put in an arm sling and was instructed not to move his arm until his first specialist outpatient review at two weeks postoperatively in order to maintain suture integrity.

At the two-week review, his wounds showed satisfactory healing with no swelling and erythema. His pain had manageable with minimal analgesia consumed on an as-needed basis. He reported no further injury to his shoulder. He was encouraged to begin physiotherapy treatment with a primary goal of starting gentle pendular movements.

During his visit to the physiotherapist at three weeks postoperatively and the specialist review at six weeks postoperatively, the patient demonstrated maximum forward flexion of 45º, extension of 30º, abduction of 45º, and external rotation of 5º.

**Figure 4:**
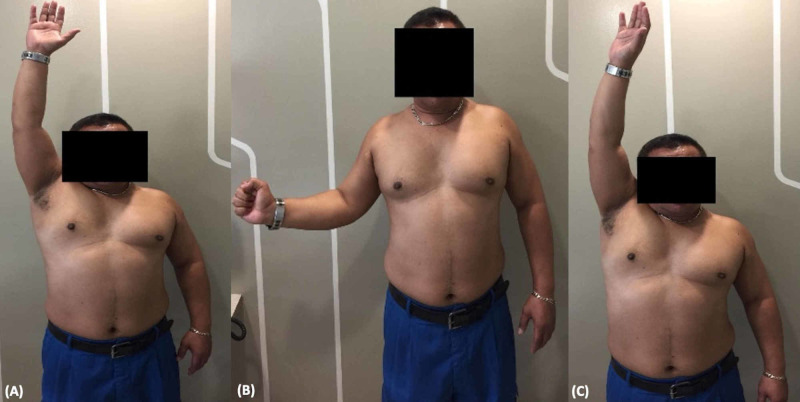
Clinical picture showing the patient demonstrating full range of motion of (A) forward flexion, (B) external rotation, and (C) abduction.

At four months postoperatively, his range of movement had improved such that his forward flexion, abduction, and external rotation were full (Figure 4). He reported no pain and was functionally independent and subjectively satisfied. Objectively, there was no weakness in the rotator cuff muscles.

## Discussion

A current paucity of evidence

This unusual case report highlights the current lack of evidence found describing concurrent full-thickness and partial-thickness RCTs, or full-thickness tear with partial-thickness extension. In preparation for this publication, an exhaustive literature search was performed, using key terms such as full-thickness tear with partial tear extension or concurrent full-thickness and partial-thickness tears, as well as numerous other iterations. It is therefore concluded that this is an extremely rare presentation of rotator cuff pathology and it is difficult to comment on the prevalence in the community. The question is also raised of the origin of the tear: whether the two tears occurred in isolation, or if a full-thickness tear occurred first with ongoing wear resulting in partial-thickness extension, or vice versa.

Diagnosis and MRI features

It is well known that pathology related to the supraspinatus has very good agreement between MRI and arthroscopic findings [9]. The supraspinatus tendon is well depicted in all coronal, axial, and sagittal planes, and all performed sequences can guide radiologists to the pathology even if some planes or sequences are missing. However, it should not be forgotten that partial-thickness tears are detected more often when using MR arthrography, especially so with the affected shoulder in the ABducted and Externally Rotated (ABER) position [10,11]. The MRI features in this case are of particular interest. It is useful to note that the patient underwent a conventional MRI and not an arthrogram. This case highlights that MRI signs of partial-thickness tears may be easy to miss when superimposed on a full-thickness tear, resulting in under-reporting of this unique presentation [12]. In this case, the only suggestion of a partial-thickness extension was an area of increased signal that suggested cystic changes. It is therefore recommended that any unexpected clues pointing towards additional pathology be explored more comprehensively especially during arthroscopy. If missed, this clinical condition may not be fully addressed at surgery, which may lead to poorer postoperative outcomes for the patient. The surgeon who performs only open repair of the rotator cuff with only a bursal-sided view is at risk of missing the partial articular-sided tear.

A hybrid approach to RCT repair

This case also highlights the need for a documented operative description that others may use as a guideline for their own management, adapting techniques of traditional partial articular-sided tendon avulsion (PASTA) approach and full-thickness tear repair.

The technical skills required to address this tear configuration should not be underestimated. To achieve stability of the repair, a considerable number and systems of sutures were required. It is hoped that the intra-operative description and arthroscopic pictures will prove fruitful to anyone else who encounters a similar presentation.

The core principles to ensure a well-healed cuff as iterated by Burkhart and Hartzler are as follows: (1) the RCT pattern must be recognized, starting with a careful assessment of preoperative MRI but concluding with the arthroscopic assessment of tear edge mobility; (2) a low-tension, anatomic, and mechanically robust repair construct must be determined based on the tear pattern; (3) the biological healing capacity of the repair site must be optimized by using meticulous preparation of the greater tuberosity bone; and (4) aggressive early rehabilitation after arthroscopic rotator cuff repair must be avoided respecting that tendon to bone healing is unlikely to occur before 12 weeks postoperatively [13].

## Conclusions

A full-thickness supraspinatus tear confluent with a separate partial articular-sided tear is a rare combination in rotator cuff pathology. Imaging may not be conclusive in diagnosing both tears. Therefore, a detailed assessment of tear morphology at diagnostic arthroscopy is crucial for diagnosis. Failure to diagnose and address this tear pattern at the time of primary surgery may result in earlier failure and a more complex revision surgery. When identified at primary surgery, a hybrid suture system can be utilized to produce a stable and dynamic repair of the rotator cuff.
